# DEMENTIA CARE IN THE LAST YEAR OF LIFE: EXPERIENCES IN A COMMUNITY PRACTICE

**DOI:** 10.1093/geroni/igac059.1828

**Published:** 2022-12-20

**Authors:** Mairead Bartley, Jennifer Manggaard, Karen Fischer, Diane Holland, Paul Takahashi

**Affiliations:** Mayo Clinic Rochester, Rochester, Minnesota, United States; Mayo Clinic, Rochester, Minnesota, United States; Mayo Clinic, Rochester, Minnesota, United States; Mayo Clinic, Rochester, Minnesota, United States; Mayo Clinic, Rochester, Minnesota, United States

## Abstract

**Objectives:**

People with dementia often have high care needs at end of life. We compared care delivery in the last year of life for people living with dementia in the community (home/assisted living facilities, ALF) to those in skilled nursing facilities (SNF).

**Methods:**

This was a retrospective study of people who died with a diagnosis of dementia in the community compared with SNF between 2013 and 2018. Primary outcomes of hospitalizations/ED visits in the last year of life were measured. Secondary outcomes included advance care planning (ACP) completion, hospice enrollment, provider visits and ICU admissions.

**Results:**

Of 1203 people with dementia, 581 (48.3%) were living in a SNF and 622 (51.7%) in the home/ALF. At least one hospitalization was recorded for 70.7% in the home/ALF compared with 50.8% in the SNF (p< 0.01), similar to ED visits (80.2% versus 58% in home/ALF and SNF groups, respectively, p< 0.01). People in the SNF had more provider visits (median 9 (IQR 6, 12) compared with 5 (IQR 3,9) in the home/ALF group (p< 0.01). There was no ACP for 12% of those in the home/ALF, compared with 4.6% in the SNF(p< 0.01). Approximately half (56.5%) in the SNF were enrolled in hospice compared with 68.3% in the home/ALF group (p< 0.01), Median time in hospice was 26.5 days in the SNF versus 30 days in the home/ALF (p=0.67).

**Conclusions:**

People living with dementia have frequent acute care utilization in the last year of life. Hospice care was more common in the home/ALF. Time in hospice was short.

